# Continuous Positive Airway Pressure Treatment Reduces Mortality in Elderly Patients with Moderate to Severe Obstructive Severe Sleep Apnea: A Cohort Study

**DOI:** 10.1371/journal.pone.0127775

**Published:** 2015-06-11

**Authors:** Qiong Ou, Yong-Chi Chen, Sheng-Qing Zhuo, Xiang-Ting Tian, Chun-Huan He, Xi-Lin Lu, Xing-Lin Gao

**Affiliations:** 1 Sleep Center, Department of Respiratory Medicine, Guangdong General Hospital, Guangdong Academy of Medical Sciences, Guangdong Provincial Institute of Geriatrics, Guangzhou, Guangdong, People’s Republic of China; 2 Department of Cardiology, Guangdong General Hospital, Guangdong Academy of Medical Sciences, Guangdong Provincial Institute of Geriatrics, Guangzhou, Guangdong, People’s Republic of China; 3 Department of Neurology, The First Affiliated Hospital, Sun Yat-sen University, Guangzhou, Guangdong, People’s Republic of China; Azienda Ospedaliero-Universitaria Careggi, ITALY

## Abstract

Obstructive sleep apnea (OSA) is much more prevalent in older people than in middle-aged or young populations, and has been associated with cardiovascular disease. Continuous positive airway pressure (CPAP) is the first-line therapy for OSA, but its long-term clinical benefit in the elderly is unclear. Here, we carried out a prospective cohort study to explore the survival rate and incidence of cardiovascular events in elderly patients with moderate to severe OSA who did or did not receive CPAP treatment. The study included 130 patients (104 male, 26 female; mean age: 77.8 ± 6.2 years) who were followed up for a mean of 5 ± 2.54 years (range, 1–8 years). Thirty-six patients received CPAP and 88 had no CPAP. The results showed that mortality in the untreated group (21.6%) was significantly higher than in the CPAP group (5.6%). Kaplan–Meier survival analysis showed that the survival rate in the CPAP group was 94.4%, which was markedly higher than the rate of 78.4% in the untreated group. The incidence of cardiovascular events was 13.9% in the CPAP group and 55.7% in the untreated group. The present study provides evidence that CPAP can reduce mortality in older patients with moderate to severe OSA, and lead to a good long-term prognosis. The study also indicates that death in older OSA patients is associated with cardiovascular disease and diabetes.

## Introduction

Obstructive sleep apnea (OSA) is a common sleep-related breathing disorder, which has been recognized as a risk factor for cardiovascular disease such as hypertension, coronary artery disease and stroke [1,2]. The morbidity related to sleep apnea in adults is 2–4%, and is higher in the elderly than that in middle-aged and young people [3,4]. However, the clinical manifestations of sleep apnea are not often obvious in the elderly [3,4], thus it is often undiagnosed. Continuous positive airway pressure (CPAP) is the first-line therapy for sleep apnea, and resolves disordered breathing during sleep, normalizes oxygen saturation, and improves nocturnal and daytime symptoms. Substantial evidence has documented that OSA can increase long term mortality in general OSA populations. However, few studies specifically address the effect of CPAP on the OSA-associated mortality in the elderly, Thus, the long-term effect of CPAP on OSA remains unclear in the elderly.

Recent research has shown a beneficial effect of CPAP on the incidence of hypertension, coronary artery disease, and chronic heart failure in middle-aged patients with OSA [5–7]. However, there have only been a few reports regarding the effects of CPAP on OSA in elderly patients, with little investigation of its long-term efficacy. In addition, some deaths during sleep in the elderly have been associated with OSA [8–10], but there is a lack of information on whether CPAP treatment can decrease the cardiovascular event rate and mortality in elderly subjects with OSA.

We report a prospective study of a cohort of elderly with moderate to severe OSA and found a beneficial effect of CPAP treatment on long-term outcomes.

## Methods

### Subjects

Elderly subjects (≥ 60 years) with moderate to severe sleep apnea (apnea-hypopnea index [AHI] ≥ 20 events/h) were recruited from the Institute of Geriatrics in the Sleep Medicine Center, Guangzhou between 1 January 1998 and 31 December 2006. The study was approved by the Ethics Committee of Guangdong General Hospital. All subjects gave written informed consent for participation of the study. The health status of the subjects varied widely but none were institutionalized and all were fully ambulatory, with subjectively good health, no history of cancer, and no acute illness at baseline.

Subjects were examined clinically by overnight polysomnography (PSG, Alice 4, Philips Respironics, Inc., Murrysville, PA, USA), electroencephalography, electro-oculography, electromyography, tibialis electromyography, electrocardiography, and measurement of respiratory effort by a respiratory belt, heart rate, oronasal airflow (thermistor), and arterial oxygen saturation by finger oximetry. Experienced technicians scored the PSG records for sleep stages and respiratory events based on the recommendations of the American Academy of Sleep Medicine Task Force (1999). A hypopnea event was defined as a reduction in airflow amplitude of > 50% from baseline lasting at least 10 s, or a less significant reduction in airflow amplitude accompanied by the presence of arousal or oxygen desaturation of at least 3%. The oxygen desaturation index (ODI) was calculated by dividing the mean number of 4% oxyhemoglobin desaturation events by hours of sleep. The AHI was calculated as the mean number of episodes of apnea and hypopnea per hour of sleep. Sleep apnea was diagnosed if the AHI was ≥ 5 events/h (mild sleep apnea was defined as AHI 5 to < 20/h, moderate sleep apnea as AHI 20 to < 30/h, and severe sleep apnea as AHI > 30/h). The medical history of each subject was obtained during a clinical interview with the patient, and from medical records.

### Treatment

CPAP treatment was recommended to patients with moderate or severe sleep apnea. An alternative intraoral protrusive appliance was offered to patients who refused CPAP or to those with mild sleep apnea without severe hypersomnolence. Patients who refused mechanical devices remained untreated.

### Follow-up

Patients underwent follow-up examinations at least annually, and these included medical history-taking, physical examination, and appropriate supplementary investigations to assess cardiovascular events. If patients had not visited the sleep laboratory within the last 6 months before the end of the study, and could not be reached by repeat telephone calls, the latest available dataset of the annual follow-up investigations was used for censoring of event-free survival. Follow-up time was counted from study entry to the latest available dataset or to the cardiovascular event. Death from cardiovascular or other diseases, acute coronary syndrome, revascularization procedures (coronary artery bypass graft, angioplasty), and stroke were determined using a standardized questionnaire in telephone calls to patients or their relatives, and from examination of hospital records. Compliance was defined as use of CPAP for at least 4 h per night on average. If the CPAP device did not have a built-in time counter, compliance reported by the patients was used.

### Endpoints

Endpoints were nonfatal and fatal death from cardiovascular events (myocardial infarction or stroke). Nonfatal events included myocardial infarction, stroke, and acute coronary syndrome requiring revascularization procedures. Only one event was allowed for each subject. Endpoint classification was based on hospital records and telephone calls with specialist physicians. Death certificates and autopsy reports, if available, were obtained for patients who died during follow-up. All patients or relatives were contacted by phone using a standardized questionnaire. The classification of myocardial infarction and stroke was made according to the discharge diagnosis after hospitalization.

### Statistical analysis

Measured data are presented as mean **±** standard deviation. All p-values reported are two-tailed, and p-values < 0.05 were considered statistically significant. Intergroup differences were analyzed for significance using the Student *t*-test for unpaired samples. A normal distribution was assumed because of the large sample size. Categorical variables were compared using Fisher’s exact test. Event-free survival curves were calculated using the Kaplan–Meier method and compared with the log-rank test. Logistic regression was used for multivariable analysis. Results of logistic regression are presented as the hazard ratio (HR) ± 95% confidence interval (CI). Statistical analyses were performed with SPSS version 13.0 (SPSS Inc., Chicago, IL, USA).

## Results

### Patient characteristics

A total of 130 patients (104 male, 26 female; mean age: 72.8 ± 6.2. years) were recruited. Six subjects (4.6%) were lost to follow-up. CPAP treatment for sleep apnea was initiated in 36 patients, and 88 patients refused treatment. Patient baseline characteristics are listed in Table 1. Briefly, AHI was higher in the treated group (45.3 ± 13.0 vs. 36 ± 13.9.0; p = 0.001), snoring was higher in the treated group (88.9% vs. 71.6%; p = 0.039), Epworth Sleepiness Score was higher in the treated group (8.1±3.5 vs. 6.1 ± 3.5; p = 0.007), and insomnia was higher in the untreated group (14.8% vs. 2.8%; p = 0.049). There were no significant differences regarding age, sex, body mass index (BMI), ODI, and preexisting cardiovascular disease.

**Table 1:** Clinical characteristics and cardiovascular features of treated *vs* untreated OSA patients at baseline.

	CPAP (n = 36)	No CPAP (n = 88)	P
**Sex, male, n (%)**	33 (91.7%)	71 (80.7%)	NS
**Age (years)**	71.25±6.31	73.39±6.05	NS
**Height (cm)**	168.58±7.72	165.77±6.63	NS
**Body weight (kg)**	71.36±11.26	70.73±10.73	NS
**BMI (kg/m** ^**2**^ **)**	30.78±9.00	30.92±8.65	NS
**Habitual snoring, n (%)**	32 (88.9)	63 (71.6)	0.039
**ESS score**	8.08 ±3.49	6.19±3.50	0.007
**ESS ≥ 9, n (%)**	21 (58.3)	51 (58.0)	NS
**Chronic insomnia, n (%)**	1 (2.8)	13 (14.8)	0.049
**Hypertension, n (%)**	29 (80.6)	55 (52.5)	NS
**Coronary heart disease, n (%)**	19 (52.8)	40 (45.5)	NS
**Diabetes, n (%)**	5 (13.9)	15 (17.0)	NS
**Stroke, n (%)**	5 (13.9)	11 (12.5)	NS
**AHI**	45.33±13.05	36.05±13.94	0.001
**Average SaO**_**2**_ **(%)**	93.19±2.51	94.04±2.26	NS
**%Time < 90% Sat**	49.98±50.54	39.38±64.23	NS
**ODI**	37.68±17.41	32.92±15.42	NS

CPAP, continuous positive airway pressure. BMI, body mass index. AHI, apnea-hypopnea index. SaO2, oxygen saturation. ESS, Epworth Sleepiness Scale. ODI, oxygen desaturation index. OSA, obstructive sleep apnea. NS, not significant.

### Mortality

Twenty-one patients died (48.8%) during follow-up. The risk of death in patients without CPAP treatment (19/88; 21.6%) was higher than in those who underwent CPAP (2/36; 5.6%) (p = 0.035). The major cause of death was cardiovascular disease (Table 2). Logistic regression analysis using death as the dependent variable and age, disease, BMI, AHI, and no CPAP as independent variables, established that no CPAP treatment, hypertension, coronary heart disease, diabetes, and AHI were significantly associated with mortality (Table 3). In the final model, no CPAP was the most significant predictor of death.

**Table 2:** Comparison of mortality and causes of death between the with/without CPAP groups.

	With CPAP	Without CPAP
**n**	36	88
**Death, n (%)**	2 (5.6)	19 (21.6)
**Cardiovascular deaths, n(%)**	2 (5.6	12 (13.6)
**Cardiac deaths, n**	1	10
**Ischemic stroke, n**	1	2
**Noncardiovascular deaths, n (%)**	0	6 (6.8)
**Infection, n**		2
**Tumors, n**		1
**Other, n**		3

AHI, apnea-hypopnea index.

**Table 3:** Multivariable analysis of mortality.

Variable	Parameter estimate	χ^2^	p	Hazard ratio	95% Confidence Interval
**Without CPAP**	2.496	0.854	0.003	12.195	2.28–64.71
**Hypertension**	1.543	0.738	0.037	4.678	1.10–19.88
**Coronary heart disease**	1.576	0.607	0.009	4.837	1.47–15.89
**Diabetes**	1.322	0.631	0.036	3.751	1.08–12.92
**AHI**	1.576	0.676	0.02	4.834	1.28–18.19

AHI, apnea-hypopnea index.

### Survival analysis

**Fig 1:**
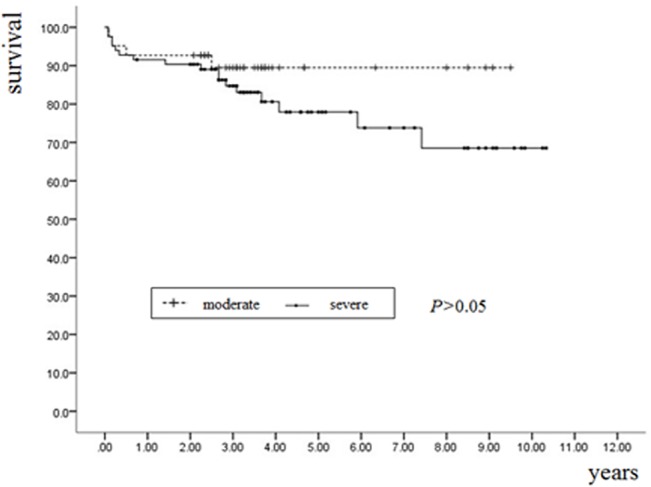
Kaplan–Meier estimates of the probability of event-free survival with or without CPAP in patients with OSA (Log-rank χ^2^ = 6.33, p = 0.01).

The median follow-up period was 5 ± 2.5 years (range, 1–8 years). The survival rate was significantly higher in those treated with CPAP than in those without CPAP (94.4%, 95%CI: 82–99% vs. 78.4%, 95%CI: 70–87%; log-rank χ^2^ = 6.3; p = 0.01). The estimated event-free survival at 5 years was 93.8% and 69.3%, respectively, in patients with and without CPAP (log-rank test: p < 0.01) (Fig 1).

**Fig 2:**
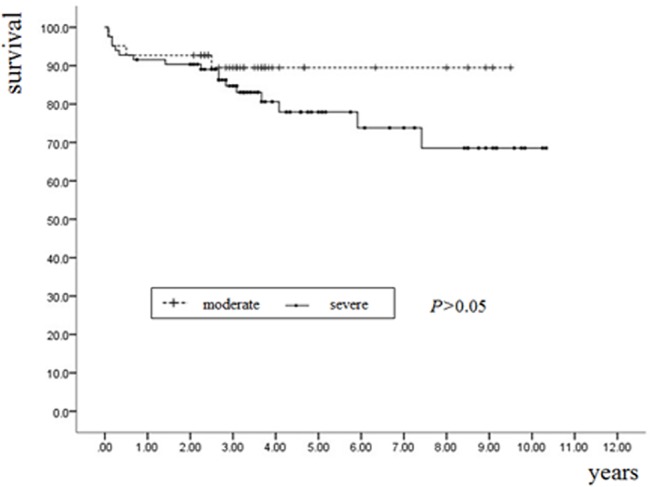
Kaplan–Meier estimates of the probability of event-free survival in Moderate or severe OSA patients (Log-rank χ^2^ = 1.506, p = 0.22).

The survival rate was 87.3% (48/55, 95%CI: 77–99%) in patients with moderate sleep apnea and 74.8% (74/99, 95%CI: 71–88%) in patients with severe sleep apnea, but this difference was not statistically significant (log-rank test, p = 0.22) (Fig 2).

### CPAP compliance and events

A total of 53 cardiovascular events occurred during the observation period in the whole study group. Cardiovascular events were more frequent in untreated than in treated patients (5 [13.9%] in treated patients vs. 48 [55.7%] in untreated patients, p = 0.000).

The minimum, maximum and mean treatment times were 2 months, 5.5 years and 2.2 ± 1.5 years, respectively. Good compliance was defined as CPAP treatment ≥ 4 h every night for more than 5 days per week, otherwise it was considered poor compliance. There was good compliance in 66.7% of CPAP patients (24/36) and poor compliance in 33.3% (12/36). The incidence of events in patients with good compliance was 4.2% (1/24), which was markedly lower than in patients with poor compliance (16.7%; p = 0.03).

## Discussion

It is generally accepted that severe forms of sleep apnea require treatment with CPAP to reduce cardiovascular risk, especially when associated with daytime sleepiness. However, the clinical significance of CPAP in elderly patients is unclear and little is known about its benefits in this age group. We therefore performed a prospective observational study to evaluate the impact of sleep apnea treatment on cardiovascular events in a cohort of elderly patients suffering from moderate to severe sleep apnea.

Campos-Rodriguez et al. studied a group of sleep apnea patients aged 55.4 ± 10.1 years, and reported that the mortalities of treated and untreated patients were 5.3% and 16%, respectively [5]. Regarding the effect of CPAP treatment on mortality, previous research has focused on the relationship between CPAP compliance and death, and only included patients who underwent CPAP [11–15]. However, most patients, especially elderly patients diagnosed with sleep apnea, did not accept treatment because of an insufficient understanding of sleep apnea and its treatment. There has been little investigation of the consequences of not treating sleep apnea in elderly patients, who are affected by sleep apnea differently from other adults because of the decline in organ functions accompanied by multiple chronic age-related diseases.

The current study showed that mortality in the untreated group was significantly higher than in the CPAP group, and was also higher compared with that of untreated sleep apnea patients in other reports. The major cause of death in our study was cardiovascular diseases, which was consistent with previous studies [16,17]. Multivariable analysis suggested that death in elderly patients with OSA was significantly associated with the absence of CPAP treatment, OSA severity and other factors at baseline, such as hypertension, coronary heart disease and diabetes. In previous reports, the relationship between OSA and cardiovascular disease, such as hypertension and coronary heart disease, was usually reported [18–20], but the correlation between mortality in OSA patients and diabetes was rarely mentioned. Our study also revealed that death in sleep apnea patients was not only associated with hypertension and coronary heart disease, but also with diabetes.

However, the most important of all the above influential factors for OSA-related death was the lack of CPAP treatment, which further demonstrates the important role of CPAP treatment in the prognosis of elderly patients with OSA.

Previous studies have reported that OSA increases the risk of cardiovascular disease [21–24], but whether CPAP treatment decreases the risk remains unknown, especially in elderly patients with cardio-cerebrovascular diseases [25–29]. Our study indicated that the incidence of cardiovascular events was 13.9% in the CPAP group and 55.7% in the untreated group, with the implication that CPAP treatment reduces cardiovascular events in elderly patients with OSA. Retrospective multivariable analysis of mortality revealed that cardiovascular events correlated with non-treatment with CPAP treatment and daytime sleepiness, which were considered as risk factors for cardiovascular events independent of age, gender, BMI and AHI in elderly patients with OSA. In the evaluation of new cardiovascular events, CPAP treatment had a long-term effect in reducing recurrence of these events, and should be taken into account when considering the clinical goals of treatment for elderly patients with moderate to severe OSA.

OSA is a common chronic disease, but how its prognosis can be evaluated is an important problem. Survival analysis is a commonly used method to assess the prognosis and effects of chronic diseases. Our results suggest that CPAP treatment could enhance the survival of elderly patients with moderate to severe OSA，The 5-year survival rate of elderly treated OSA patients was 93.8%, but the 5-year survival rate of elderly untreated OSA patients was 69.3%, which was lower than that reported for untreated adult patients with OSA (77.9%) [5].

Although our data suggests that CPAP group has less mortality than the untreated OSA group, it should be very cautious to interpret this result. The association of CPAP with low mortality may not be necessarily causal due to the observational design. We could not completely rule out the possibility that the other factors rather than CPAP may also contribute to the reduction of the mortality in CPAP group. Nevertheless, CPAP patients had higher AHI and more sleepiness than the non-CPAP patients, indicating the beneficial effects of CPAP on elderly patients with OSA. Additionally, the sample size of the study is small. Whether sleep apnea can be considered as an independent risk factor for mortality in elderly patients requires further analysis in randomized trials with larger groups of treated and untreated sleep apnea patients.

## Conclusion

This study indicates that there is a benefit of long-term CPAP treatment in elderly patients with moderate to severe sleep apnea. CPAP treatment was associated with a marked reduction in the risk of death and cardiovascular events independent of age, gender, BMI and preexisting cardiovascular comorbidities. However, because there was less than optimal compliance with this treatment, there is a need for more studies that focus on improving the adherence to and tolerance of CPAP treatment in these patients.
