# Correction: Continuous Positive Airway Pressure Treatment Reduces Mortality in Elderly Patients with Moderate to Severe Obstructive Severe Sleep Apnea: A Cohort Study

**DOI:** 10.1371/journal.pone.0201923

**Published:** 2018-08-01

**Authors:** Qiong Ou, Yong-Chi Chen, Sheng-Qing Zhuo, Xiang-Ting Tian, Chun-Huan He, Xi-Lin Lu, Xing-Lin Gao

Fig 1 is a duplicate of Fig 2. The authors have provided a corrected version of Fig 1 here.

**Fig 1:**
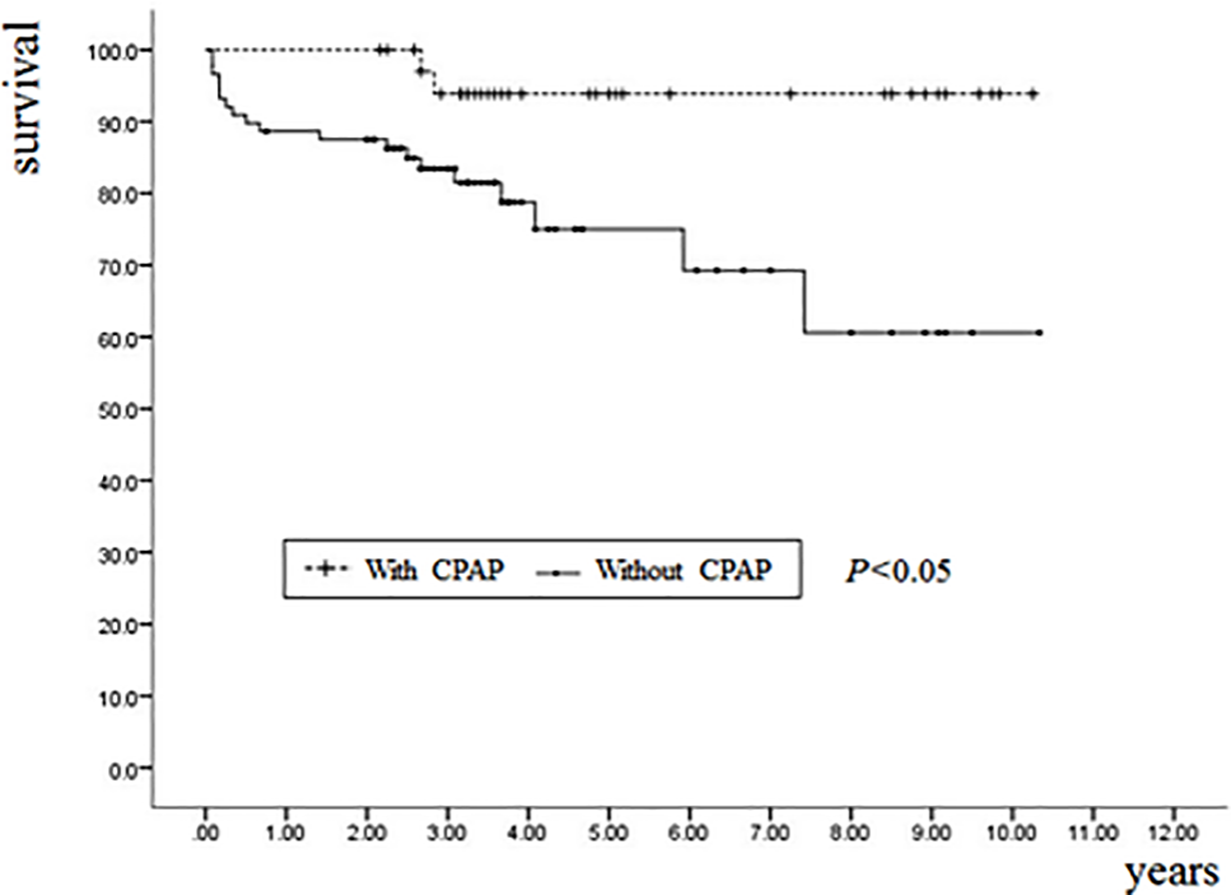
Kaplan–Meier estimates of the probability of event-free survival with or without CPAP in patients with OSA (Log-rank χ2 = 6.33, p = 0.01).
